# Explanatory factors for the survival benefit among hip and knee arthroplasty patients with osteoarthritis

**DOI:** 10.1016/j.ocarto.2025.100587

**Published:** 2025-02-22

**Authors:** Jan M. Heijdra Suasnabar, Maaike Gademan, Liza van Steenbergen, Ewout Steyerberg, Rob Nelissen, Wilbert van den Hout

**Affiliations:** a Department of Biomedical Data Science, Leiden University Medical Center, Leiden, the Netherlands; b Department of Orthopedics, Leiden University Medical Center, Leiden, the Netherlands; c Department of Clinical Epidemiology, Leiden University Medical Center, Leiden, the Netherlands; d Dutch Arthroplasty Register (LROI), ‘s-Hertogenbosch, the Netherlands

**Keywords:** Osteoarthritis, Arthroplasty, Mortality, Socioeconomic status, Quality of life

## Abstract

**Objective:**

Studies have shown that osteoarthritis patients who underwent a primary total hip or knee arthroplasty (THA/TKA) experience better survival than the general population, yet there is limited evidence explaining this counter-intuitive difference. We investigated whether this better survival is also present in the Netherlands and to what extent it could be explained by a patient selection effect, whereby patients with more favorable health and socioeconomic status (SES) are more likely to receive THA/TKA.

**Design:**

In this registry-based study, we compared the survival, health and SES of THA/TKA osteoarthritis patients to those of the general Dutch population. The patient cohort included 224,785 THA and 198,691 TKA patients who underwent an arthroplasty between 2010–2020. The proportions of the survival differences explained by better health (as measured by the EQ-5D) and SES (postcode-level) were estimated using spline-based survival models and Dutch lifetables.

**Results:**

The eleven-year survival of THA and TKA patients were 8.7% and 8.1% better than the general population. Although health and SES predicted individual survival, they explained only ≈7% of the survival benefit.

**Conclusions:**

Our study confirmed that Dutch osteoarthritis THA/TKA patients experience better survival than the general population, but raises important questions as to the explanation. A more favorable health status and/or SES did not explain most of the survival benefit. This may be partly due to limitations of the available measures of health and SES in our study, but also leaves other explanations (e.g. barriers to receive access to care, lifestyle changes) open for further research.

## 1 Introduction

Studies in several countries have shown that survival of osteoarthritis (OA) patients after total hip and knee arthroplasty (THA/TKA) is better than the survival in the general population matched on gender, age, and calendar year [[1], [2], [3], [4], [5]]. However, this apparent post-arthroplasty benefit is not universally observed and it is unclear which factors may explain it [6,7]. Although arthroplasty may improve survival among OA patients compared to OA patients without arthroplasty, we would not expect such improvements to reach a level whereby a survival benefit compared to the general population is observed [7]. A more likely hypothesis is that a selective group of patients receives arthroplasty, i.e., a selection of patients with otherwise more favorable life expectancy than the general population.

Different processes could lead to a survival benefit among patients receiving arthroplasty. Physicians may be more likely to offer arthroplasty to patients who are otherwise (i.e. if it were not for the OA) healthier than the general population, resulting in above-average life expectancy. Alternatively, patients with lower life expectancy may prefer not to undergo the procedure or may have difficulty accessing it. Indeed, lower arthroplasty rates have been reported among older patients and those from poorer socioeconomic backgrounds [8]. Underrepresentation of older patients would not result in better survival compared to a gender-and-age-matched general population, but it would do so if it especially applies to older patients with poorer health.

Alternatively, an overrepresentation of patients with above-average socioeconomic status (SES) could contribute to the survival benefit. Although access to healthcare in the Netherlands is relatively equitable with minimal financial barriers, there are still large differences in health depending on SES [9,10]. Besides access to care, SES is also related to individual preferences, values, and lived experiences, which in turn influence decision making regarding arthroplasty [11]. As OA is associated with a high burden of disease, lower arthroplasty rates among patients with lower SES may underlie existing health inequalities. Identifying such inequalities among THA and TKA patients could start a process towards better preoperative consultation and more equitable healthcare in the Netherlands.

This study aimed to confirm whether a survival benefit among OA patients who underwent elective THA and TKA is also present in the Netherlands, and to estimate to what extent it can be explained by selection on health status and SES of patients.

## 2 Methods

### 2.1 Study population and data sources

We retrospectively analyzed data of patients with OA who underwent a primary THA (*N* ​= ​224,785) or TKA (*N* ​= ​198,691) between January 1, 2010 and December 31, 2020 in the Netherlands, as registered in the Dutch Arthroplasty Register (LROI). The coverage of THAs/TKAs in the LROI was 99% in 2020 [12]. Hip and knee prosthesis patients with a diagnosis of OA were included if they were 18–100 years old at operation and had complete data on (at least) sex, age, survival time/status, and year of operation. If patients underwent a second contralateral procedure in the same type of joint, that second procedure was excluded. In the case of simultaneous bilateral procedures (recorded as two entries in the LROI) only one entry was included to ensure one entry per patient.

The following patient reported outcome measures at baseline (i.e. the time of the THA/TKA procedure) and 12 months post-procedure were included: the EuroQol 3-level Quality of Life Questionnaire (EQ-5D) [13], the EuroQol Quality of Life Visual Analogue Scale (EQ-VAS), the Hip/Knee Disability and Osteoarthritis Outcome Score (HOOS-PS/KOOS-PS, for THAs/TKAs respectively) [14,15], and the Oxford Hip/Knee Score (OHS/OKS, for THAs/TKAs respectively) [16]. Additional data recorded at baseline included: body-mass index, type of hospital/clinic (general/university/focus clinic), postcode SES, American Society of Anesthesiologists (ASA) Classification [17], and Charnley classification [18]. The SES variable was a standardized score based on patients’ 4-digit postcode calculated by the Netherlands Institute for Social Research based on levels of income, education, and unemployment in each postcode area in 2017 (mean SES in the Netherlands ​= ​−0.138, SD ​= ​1.198) [19].

### 2.2 Generic health without osteoarthritis

Patients described their health status using the three-level EQ-5D, from which we calculated Dutch utility scores [20]. Utility scores reflect the value of health, anchored at 1 (perfect health) and 0 (as bad as dead). This observed overall utility includes the burden of OA and is denoted here by $U_{inc}$. To test the hypothesis that our study sample was otherwise (i.e. besides the OA) healthier than the general population, we estimated a measure of health utility $U_{ex}$ that excluded the burden of OA. We refer to this non-OA related utility as patients’ *generic health*. For a fair comparison with general population utilities, such a measure of generic health must only exclude the burden due to OA but, crucially, should still include information on any other circumstances such as a comorbidity or good health. Therefore, to estimate $U_{ex}$ we assumed a multiplicative model [21,22], where OA-related and non-OA related (e.g., a comorbidity) utility have a combined multiplicative impact on the overall utility:

$$U_{ex}=\frac{U_{inc}}{U_{\text{OA}}\left(X\right)}$$

here $U_{\text{OA}}\left(X\right)$ represents the unobserved utility if the patient would only have OA, dependent on the burden of OA as described by a set of variables X. We estimated this OA-related utility using response mapping [23]. Specifically, we used generalized ordered logit models to estimate probabilities for patients' responses to the five EQ-5D dimensions, as functions of patients’ HOOS/KOOS scores, OHS/OKS scores, sex, and age at operation [23,24]. As noted earlier, $U_{\text{OA}}\left(X\right)$ only captures OA-related variations in utility and ignores any variations that would arise from other circumstances (e.g., a comorbidity or good health), hence the need for the multiplicative model in the formula for $U_{ex}$. More details about the calculation of $U_{ex}$ and $U_{\text{OA}}\left(X\right)$ are presented in Supplementary Appendix 1.

As primary analysis to estimate generic health without osteoarthritis, we applied the described procedure to the EQ-5D and X data at 12-months. By that time part of the burden due to osteoarthritis would already be removed by the arthroplasty, leaving less burden to be removed statistically. In three secondary analyses we alternatively used EQ-5D at baseline, EQ-VAS at baseline, and EQ-VAS at 12-months (more details in Supplementary Appendix 1).

### 2.3 Statistical analyses

Analyses were conducted separately by sex, type of procedure (THAs/TKAs) and, where relevant, age group. To handle missing data, multiple imputation (MI) was used to generate 50 complete MI datasets per type of procedure [25]. The MI models included all PROMS at 0 and 12 months, sex, age, ASA and Charnley categories, BMI, type of care provider, and SES. The percentage of missing data for patient reported outcome measures is reported in Supplementary Table S1.

The cumulative relative survival (RS) of patients was estimated as:

$$\text{RS}=\frac{S\left(t\right)}{S^{*}\left(t\right)}$$

where S(t) is the observed cumulative survival of patients over follow-up and $S^{*}\left(t\right)$ is the survival of the Dutch general population matched on sex, age, and calendar year [26].

To determine whether THA/TKA patients had a more favorable socioeconomic and/or health status than the general population, their measures were compared to population norms using t-tests. Patients' mean SES scores were compared to the Dutch mean SES score of −0.138 (SD ​= ​1.198). Patients’ estimated generic health status (i.e., $U_{ex}$) was compared to general population norms of the EQ-5D available by sex and age, including standard errors which were used for sex-and-age weighted comparisons [27].

The influence of SES and generic health on survival among THA and TKA patients was estimated using a flexible (i.e., spline-based) parametric survival model [28,29]. The number of internal knots was determined by comparing the Akaike Information Criterion of the baseline models with 2–4 internal knots [30], of which 3 knots produced the lowest Akaike Information Criterion. Covariates in the survival model were SES, generic health status and their interaction (if statistically significant). To account for age during follow-up, the models were fitted using two timescales: time since operation and attained age, where the second timescale is modelled as a function of the first timescale using age at operation as the starting age [31,32].

Differences between expected and predicted life expectancy were used to estimate the extent to which SES and generic health explained the better survival. Predicted life expectancy for each individual was calculated as the area under their extrapolated survival curves from the survival model predictions. Expected life expectancy for each individual was extracted from Dutch lifetables matched on sex, age, and year [33]. Thus, the explained percentage of the difference in survival (denoted by EPD) by SES and health was estimated as:

$$\text{EPD}=\frac{{{LE}_{\text{LROI}}-LE}_{\text{LROI}}^{*}\;}{{LE}_{\text{LROI}}-{LE}_{\text{NL}}}\times 100\%$$

where ${LE}_{\text{LROI}}$ equals the patients' average life expectancy at observed values of SES and health, ${LE}_{\text{LROI}}^{*}$ is the patients' life expectancy adjusting for the differences in SES and health between patients and the general population (i.e., assuming predictor values to equal general population norms), and ${LE}_{\text{NL}}$ is the patients’ expected life expectancy according to Dutch life tables [33]. When there was no excess survival to be explained (i.e. ${LE}_{\text{LROI}}\leq {LE}_{\text{NL}}$) the EPD was set at 0%.

## 3 Results

The THA and TKA cohorts were comparable (Table 1) in terms of sex distribution (≈65% female), age at operation (≈69 years), OHS/OKS scores (≈23), and HOOS-PS/KOOS-PS scores (≈50). In both cohorts, females were somewhat older than males and had poorer OHS/OKS and HOOS/KOOS baseline scores.

**Table 1:** Characteristics of THA and TKA cohorts.

	THA cohort		TKA cohort	
	Females	Males	Females	Males
	*N* ​= ​147,506	*N* ​= ​77,279	*N* ​= ​126,923	*N* ​= ​71,768
Age at operation (mean [SD])	70.4 (9.6)	68.0 (10.0)	69.2 (9.2)	67.5 (8.9)
Median follow-up years (IQR)	4.9 (2.5–7.6)	4.6 (2.2–7.2)	5.1 (2.7–7.7)	4.7 (2.5–7.3)
Baseline EQ-5D (mean [SD])	0.53 (0.27)	0.59 (0.25)	0.57 (0.26)	0.63 (0.23)
12-month EQ-5D (mean [SD])	0.84 (0.16)	0.87 (0.15)	0.82 (0.17)	0.85 (0.15)
Baseline EQ-VAS (mean [SD])	63.9 (20.0)	66.8 (19.7)	66.3 (19.4)	69.2 (19.1)
12-month EQ-VAS (mean [SD])	75.0 (18.5)	76.9 (18.2)	73.7 (18.2)	75.6 (17.9)
Socioeconomic status (mean [SD])	−0.081 (1.1)	−0.031 (1.1)	−0.181 (1.1)	−0.134 (1.1)
ASA classification (%)				
1	18.1 ​%	21.7 ​%	13.9 ​%	18.0 ​%
2	66.8 ​%	60.8 ​%	69.2 ​%	64.8 ​%
3–4	15.1 ​%	17.5 ​%	16.9 ​%	17.3 ​%
Body-mass index (mean [SD])	27.2 (4.8)	27.6 (4.2)	29.9 (5.2)	28.9 (4.5)
Provider type (%)				
General hospital	90.5 ​%	89.2 ​%	88.1 ​%	87.8 ​%
University hospital	2.4 ​%	2.7 ​%	2.2 ​%	2.3 ​%
Focus clinic	7.1 ​%	8.1 ​%	9.8 ​%	12.9 ​%
OHS/OKS (mean [SD])	22.0 (8.4)	24.2 (8.4)	21.8 (7.4)	24.6 (7.4)
HOOS-PS/KOOS-PS (mean [SD])	50.4 (17.7)	46.9 (17.3)	53.1 (15.1)	49.6 (15.0)

### 3.1 Relative survival (RS)

**Fig. 1:**
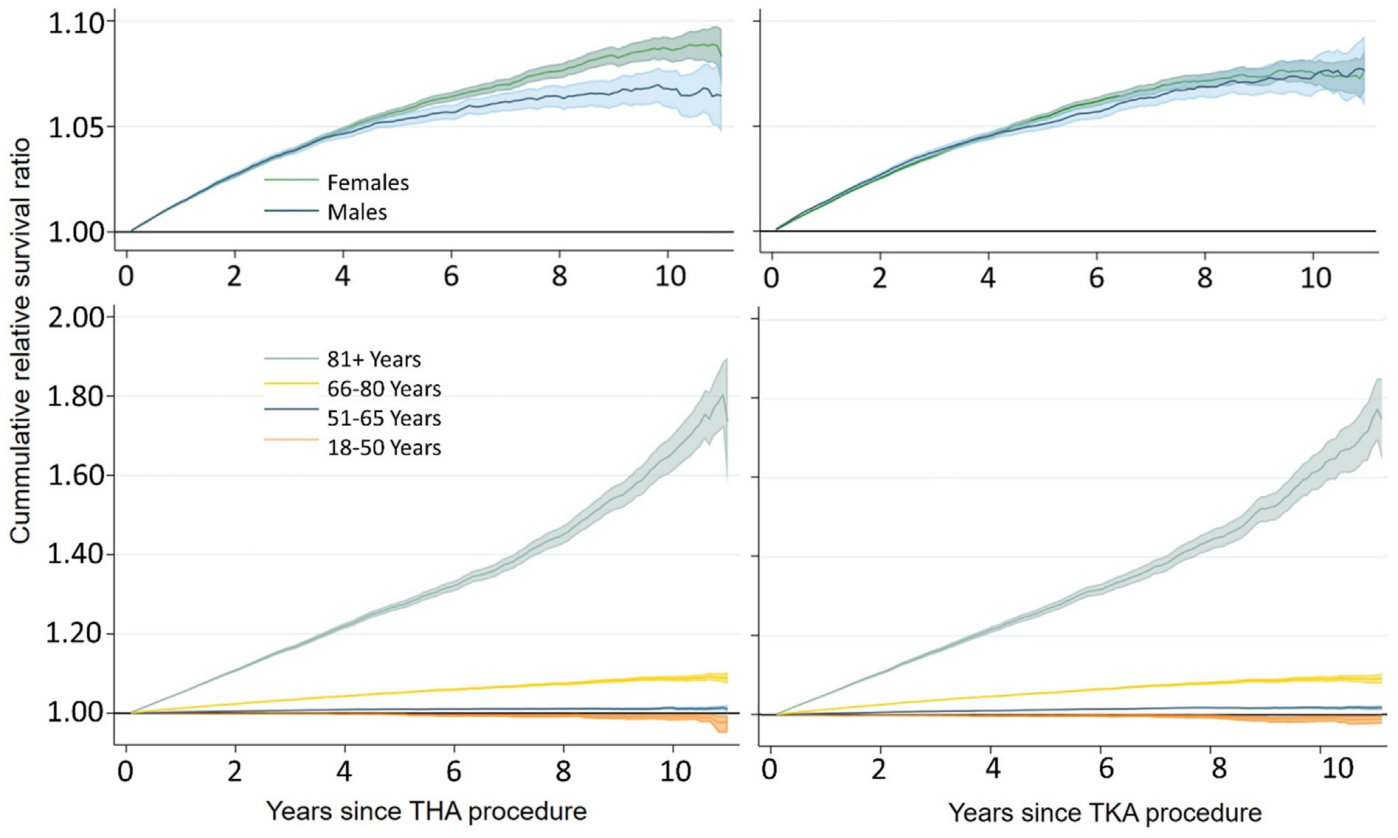
Relative survival of THA (left) and TKA (right) patients by sex and by age.

Overall, the cumulative survival of THA and TKA patients were 8.7% (RS ​= ​1.087, 95% CI: 1.080–1.094) and 8.1% (RS ​= ​1.081, 95% CI: 1.074–1.088) better than the general population survival at 11 years post-procedure. This survival benefit was observed amongst males and females (Fig. 1), and was concentrated in the two oldest age groups. The patterns in Fig. 1 are consistent with the sex-and age-stratified survival proportions at 5 and 11 years, reported in Supplementary Table S2. The only age group that experienced a similar survival relative to the general population (i.e., no survival benefit) were 18–50 year-olds.

### 3.2 SES comparisons

**Fig. 2:**
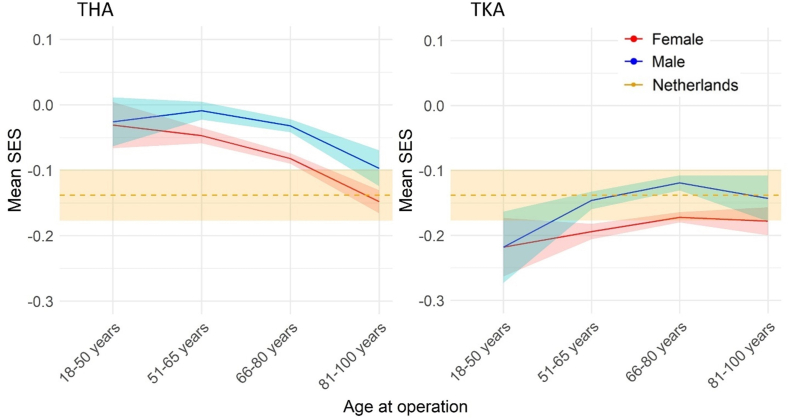
SES scores by sex and age, compared to Dutch general population.

Overall, THA patients had a slightly more favorable SES (Fig. 2) compared to the general population (mean difference ​= ​0.074, 95% CI: 0.034 to 0.114, Supplementary Table S3). In contrast, the SES of TKA patients was similar – and in some subgroups less favorable – than the general population (Fig. 2). Males consistently had a higher SES than females in both cohorts.

### 3.3 Health status comparisons

**Fig. 3:**
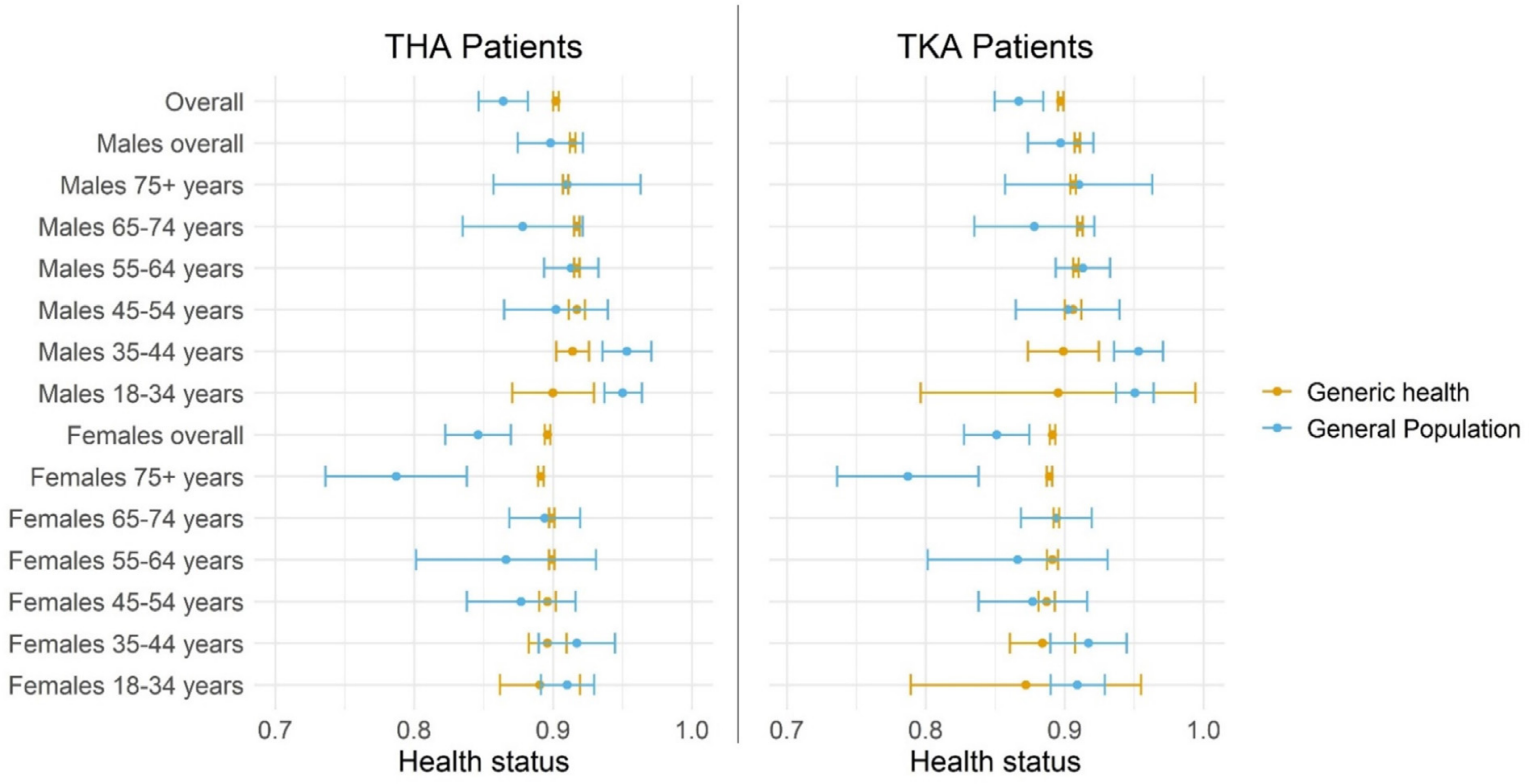
Patients' generic health status compared to the Dutch general population.

The generic health of THA and TKA patients was significantly higher than that of the Dutch general population (Fig. 3) in the primary analysis. These differences were largely due to older patients having an above-average health status.

The primary analysis results were consistent with one of the three secondary analyses, i.e., when generic health was calculated from patients' 12-month EQ-VAS (Supplementary Table S4). In the secondary analysis where generic health was calculated from patients' baseline EQ-VAS, it was only slightly higher than the general population in the TKA cohort (Supplementary Table S5). Finally, when generic health was calculated from patients’ baseline EQ-5D data, it was significantly lower than that of the general population (Supplementary Table S6).

### 3.4 Influence of SES and health status on difference in life expectancy

The overall LEs of patients were 18.7 years (THA patients) and 18.2 years (TKA patients). Matching on sex, age, and year, patients’ expected LEs according to general population lifetables were 16.9 years (THA) and 17.6 years (TKA). Better generic health and higher SES were both associated with longer survival in all flexible parametric survival models. No significant interactions were identified between SES and health in the survival models.

Adjusting patients' SES and generic health to general population norms explained 6.9% (THAs) and 6.7% (TKAs) of their survival benefit relative to the general population (Table 2). In the secondary analysis where generic health was estimated based on patients’ 12-month EQ-VAS, these percentages were 7.8% and 11.7% for THAs and TKAs patients respectively (Supplementary Table S7). Supplementary Fig. 1A–1D shows the survival of patients according to Dutch lifetables as well as their predicted survival with and without adjusting SES and health status to population norms.

**Table 2:** Percentage of survival difference explained by adjusting SES and health status to general population norms.

	THA cohort				TKA cohort			
	Difference between ${LE}_{LROI}$ and ${LE}_{NL}$ (years)	EPD by SES	EPD by Health	EPD by SES & Health	Difference between ${LE}_{LROI}$ and ${LE}_{NL}$ (years)	EPD by SES	EPD by Health	EPD by SES & Health
Overall	1.76	1.80 ​%	5.11 ​%	6.90 ​%	0.65	0.00 ​%	7.43 ​%	6.68 ​%
Males								
18–50 years	−0.17	0.0 ​%	0.00 ​%	0.00 ​%	−2.05	0.00 ​%	0.00 ​%	0.00 ​%
51–65 years	1.06	5.5 ​%	4.33 ​%	9.78 ​%	−0.11	0.00 ​%	0.00 ​%	0.00 ​%
66–80 years	1.60	2.5 ​%	4.60 ​%	7.10 ​%	1.26	0.69 ​%	3.94 ​%	4.62 ​%
81–100 years	1.92	0.7 ​%	0.00 ​%	0.18 ​%	2.17	0.13 ​%	0.00 ​%	0.00 ​%
Overall males	1.36	3.2 ​%	3.86 ​%	7.08 ​%	0.71	0.44 ​%	4.10 ​%	4.52 ​%
Females								
18–50 years	−0.02	0.00 ​%	0.00 ​%	0.00 ​%	−3.17	0.00 ​%	0.00 ​%	0.00 ​%
51–65 years	1.32	3.04 ​%	5.81 ​%	8.83 ​%	−0.71	0.00 ​%	0.00 ​%	0.00 ​%
66–80 years	2.19	1.05 ​%	5.05 ​%	6.09 ​%	1.15	0.00 ​%	5.37 ​%	4.84 ​%
81–100 years	2.66	0.06 ​%	6.79 ​%	6.84 ​%	2.47	0.00 ​%	4.98 ​%	4.75 ​%
Overall females	1.97	1.28 ​%	5.56 ​%	6.83 ​%	0.61	0.00 ​%	9.61 ​%	8.09 ​%

EPD: Explained percentage of the survival difference. SES: socioeconomic status. ${LE}_{LROI}$: life expectancy of patients without adjusting for SES or health. ${LE}_{NL}$: life expectancy according to general population lifetables.

Stratified by age and sex, adjusting patients SES and health to general population norms explained between 0% and 9.6% of their survival benefit in the primary analysis (Table 2) and 0%–70% in the secondary analyses (Supplementary Tables S7–S9). The latter 70% was due to the youngest female subgroup (THA) having a more favorable SES and health than the general population but only a small survival benefit to be explained in one of the secondary analyses (Supplementary Table S7). This was not seen in the primary analysis. Finally, despite older patients experiencing the highest survival benefit (Fig. 1) most of that benefit was not explained by a more favorable SES or generic health relative to population norms (Table 2).

## 4 Discussion

Our results confirmed that the survival of Dutch osteoarthritis THA and TKA patients was significantly better than that of the general population up to 11 years postoperatively. We also found that while more favorable SES and health are both associated with better patient survival, they only explain a small portion of the cohort's overall survival benefit relative to the general population. The survival benefit among OA patients when compared to the general population has commonly been attributed to patient selection effects, whereby patients selected for the procedures are thought to be otherwise healthier (and therefore have a better life expectancy) than the age-and sex-matched general population [7]. Ours is one of few studies to have investigated this empirically, and our findings do not support the argument that patient selection effects on health or SES are the primary explanatory factors.

In line with findings from Sweden, Norway, USA, and the UK [1,[3], [4], [5],^34^], our results confirm that the survival of THA/TKA patients with OA is also better than that of the general population in the Netherlands, and that the survival benefit was concentrated among older patients. For comparability with the referenced studies that only reported standardized mortality ratios, Supplementary Fig. S2 contains standardized mortality ratios over follow-up in this study. Interestingly, in Sweden, the survival of THA patients with OA was about 5% higher than the Swedish general population in the eighth postoperative year and then began to decline [1]. While in our study, the highest RS were higher, occurred later, and showed virtually no decline at the end of follow-up (11 years). Such differences between studies may be due to, among others, the different study periods, as follow-up in our study started about a decade later than former studies [1,3].

We further sought to determine whether a more favorable SES and/or health status among THA/TKA patients may explain their better survival. With regard to health, the study cohorts were overall marginally healthier than the general population, except for females above 75 years who were notably healthier than the age-and sex-matched general population. Adjusting patients’ health status to general population norms explained 5.1% and 7.4% of the overall differences in survival (Table 2). In subgroups without a more favorable health status than the general population (e.g. males 81–100 years), adjusting of health status did not explain any of the survival benefit. These findings show that a selection effect on health may only be present in some subgroups and even then explains a small portion of the survival benefit.

A selection effect on health among older patients (especially females) can reflect an over-representation of healthy patients and/or an under-representation of patients in poorer health. While the exclusion of patients in poor health could be reasonable, since patients with poor health are more frail and may run a higher risk of complications, further studies should explore whether more resources/efforts could be directed towards identifying/preparing older patients with a health status comparable to that of their general population, or older patients who are currently not eligible for THA/TKA due to their health but may be ‘healthy enough’ to undergo the procedure if given the appropriate pre-and post-operative care.

With regard to SES, our findings showed a less pronounced selection effect (compared to our findings on health) that was also limited to few subgroups. While the overall SES of THA patients was significantly higher than the Dutch population, that was not the case for the TKA cohort. The finding that the oldest patients, who despite experiencing the highest relative survival benefit, did not have an above-average SES, indicates that selection on SES is unlikely for this subgroup. This was also reflected by the low proportions of the difference in survival explained by SES (Table 2). On the other hand, a selection effect on SES was present among 51-65 year-old THA patients, who were better off than the general population. This may reflect a need to improve access to THAs among younger patients of lower SES, who may also be disproportionately affected by OA [35]. Future studies should also investigate ‘why’ (i.e., by what process/mechanism) Dutch OA patients with lower SES are under-represented in the THA population and over-represented in the TKA population. The Dutch healthcare system is considered to be among the most equitable, yet our findings about the SES of the THA population may suggest otherwise.

The intriguing finding that the survival benefit was not mostly explained by health and SES in this study raises the question of ‘what other factors may be attributable’. The survival benefit should probably be viewed as the result of a combination of determinants, each explaining part of the survival benefit. One such determinant may be an earlier detection of other diseases during pre-operative evaluation and/or post-operative monitoring, although we found no evidence or literature supporting this claim. Moreover, an indirect causal effect of successful THA/TKA (e.g., through recovery-related lifestyle changes, prosthesis-induced mobility, reduced pain, and increased physical activity) should not be ruled out as explanation [36,37]. The temporal trend in the relative survival, particularly among older patients (Fig. 1) may support this theory as it suggests that the benefit arises or strengthens in the later years post-arthroplasty. Currently, robust and consistent evidence on effect of arthroplasty on physical activity levels and sedentariness is lacking, especially findings from long-term studies (i.e., follow-up longer than 2 years) on populations older than 65 years old [38,39]. Finally, we note that the low differences in survival explained by more favorable SES and/or generic health (Table 2) reported in our study may be partly due to its limitations, and that other measures of SES and health may still be important explanatory factors for the survival benefit.

### 4.1 Limitations

First, the potentially biasing effect (usually towards the null) of using geographical SES as a proxy for individual-level SES is a known limitation in survival and registry-based studies such as this one [40]. Postcode-based SES measures/proxies have been shown to underestimate individual-level SES [41]. If that is the case in our study, then it is possible that SES and its ability to explain the survival benefit were underestimated.

Second, patients' measure of generic health excluding the influence of OA was unobserved because all patients actually did have OA. To estimate patients’ utility excluding the influence of OA, we used established response mapping methods [[21], [22], [23]]. However, the degree to which our estimates of generic health successfully exclude the influence of OA is limited by how well the OHS/OKS and HOOS-PS/KOOS -PS capture the full burden (and nothing but the burden) of OA. Indeed, the accuracy of our generic health estimates would be undermined if OA influences health in ways which are not associated with the OHS/OKS and HOOS-PS/KOOS-PS measures. The reverse is also true: if the OHS/OKS and HOOS-PS/KOOS-PS contain items that are not exclusively related to OA, then these items should not be used to estimate the burden of OA. To address the latter, we excluded any generally phrased items which did not explicitly attribute symptoms to OA (e.g. OHS/OKS item “Could you do the household shopping on your own?“).

Third, our reported EPD estimates (Table 2) were not high and may be subject to variability. However, given our large sample (even within subgroups), most of the variability in these EPD estimates would be attributable to their between-imputation variability [44]. We calculated the between-imputation standard deviations of these estimates and found them to range between 0% and 2.5% (in absolute scale), reflecting reasonable/good stability.

Finally, the EQ-5D was the only available measure of health status for which general population norms are also available. However, the available population norms for adults above 50 years are not very precise and may also be subject to a healthy selection effect [42], which would attenuate our reported differences in health status and its ability to explain the survival benefit. Additionally, the EQ-5D was not developed for clinical diagnostic purposes nor for survival risk prediction. Information about the presence and severity of comorbidities in the general population, such as the ASA classification or disease prevalence, would enable alternative comparisons of health status. For instance, we know that Dutch THA patients are somewhat healthier than THA cohorts in other countries in terms of ASA classification [43], however, we were unable to use ASA classification because general population distributions are unavailable.

## 5 Conclusion

Our findings confirm that THA and TKA osteoarthritis patients have a better survival than the age-and sex-matched general population and that this survival benefit is concentrated among older patients. As potential explanatory factors, we found that a selection effect on SES and health is present in some subgroups but is not able to explain much of the survival benefit. This could either be due to limitations of the health and SES measures used in this analysis, or to other determinants of (relative) survival. Further studies should jointly consider a broader range of determinants, such as lifestyle/activity changes [36,37] or access to care, that could explain the survival difference.

## Author contributions

Conceptualization: JHS, WvdH, MG, RN, ES; Data curation: JHS, WvdH, LvS; Formal analysis: JHS, WvdH; Writing - original draft: JHS, WvdH; Writing - review & editing: JHS, WvdH, LvS, MG, ES, RN.

## Ethical approval

The Medical Research Ethics Committee Leiden—Den Haag—Delft exempted this study from review under the Medical Research Involving Human Subjects Act as it did not apply to the current study (number: D4-2022-003).

## Data statement

The data analyzed in this study may not be publicly shared and is subject to a Data Sharing Agreement. The data may be made available upon request via, and at the discretion of, the Dutch Arthroplasty Register (LROI).

## Role of the funding source

This study was funded by the Netherlands Arthroplasty Register (in Dutch: Landelijke Registratie Orthopedische Interventies, LROI) as project number RG 2021–005. The funder had no influence on the design of this study, analysis and interpretation of data.

## Declaration of competing interest

All authors declare no competing interests.
