# Supplementary material for: Explanatory factors for the survival benefit among hip and knee arthroplasty patients with osteoarthritis
Source: Osteoarthr Cartil Open. 2025 Feb 22;7(2):100587. doi: 10.1016/j.ocarto.2025.100587 (PMC11925170; doi:10.1016/j.ocarto.2025.100587)

Supplementary materials

Explanatory factors for the survival benefit among hip and knee arthroplasty patients with osteoarthritis

Contents

[Supplementary appendix 1. Estimating health status without osteoarthritis 2](#_Toc178067717)

[Table 1. Percentages of missing/imputed data for PROMS 5](#_Toc178067718)

[Table 2. Five and eleven-year cumulative relative survival after THA and TKA, by sex and age group 6](#_Toc178067719)

[Table 3. Socioeconomic status scores by sex and age, compared to Dutch general population mean 7](#_Toc178067720)

[Table 4. Health status of patients compared to the general population in secondary analysis using patients’ 12-month EQ‑VAS to estimate their generic health, mean (SE) 8](#_Toc178067721)

[Table 5. Health status of patients compared to the general population in secondary analysis using patients’ baseline EQ‑VAS to estimate their generic health, mean (SE) 9](#_Toc178067722)

[Table 6. Health status of patients compared to the general population in secondary analysis using patients’ baseline EQ‑5D to estimate their generic health, mean (SE) 10](#_Toc178067723)

[Table 7. Proportions of survival difference explained by calibration of SES and generic health values to general population norms, in secondary analyses using patients 12-month EQ‑VAS to estimate generic health 11](#_Toc178067724)

[Table 8. Proportions of survival difference explained by calibration of SES and generic health values to general population norms, in secondary analyses using patients baseline EQ‑5D to estimate generic health 12](#_Toc178067725)

[Table 9. Proportions of survival difference explained by calibration of SES and generic health values to general population norms, in secondary analyses using patients baseline EQ‑VAS to estimate generic health 13](#_Toc178067726)

[Figure 1A. Predicted and expected survival of female patients after THA procedure 14](#_Toc178067727)

[Figure 1B. Predicted and expected survival of male patients after THA procedure 14](#_Toc178067728)

[Figure 1C. Predicted and expected survival of male patients after TKA procedure 15](#_Toc178067729)

[Figure 1D. Predicted and expected survival of female patients after TKA procedure 15](#_Toc178067730)

[Figure 2A. Standardized mortality ratio (SMR) over follow up (in years) among TKA patients 16](#_Toc178067731)

[Figure 2B. Standardized mortality ratio (SMR) over follow up (in years) among THA patients 16](#_Toc178067732)

# Supplementary appendix 1. Estimating health status without osteoarthritis

1. Objective

Utility scores reflect the value of health, anchored at 1 (perfect health) and 0 (as bad as dead). Consider an observed all-inclusive health utility measure $U_{inc}$:

$U_{inc}=$ Utility as is, including the impact of OA and of any other circumstances.

Other circumstances could be comorbidities, but could also be good health if the person would otherwise be healthy. This utility $U_{inc}$ as is may be regarded as the combined result of two unobserved utilities $U_{ex}$ and $U_{\text{OA}}\left( X \right)$, where

$U_{ex}=$ Utility if the person would *only* have the comorbidity (i.e. excluding the impact of OA).

$U_{\text{OA}}\left( X \right)=$ Utility if the person would *only* have OA (i.e. excluding any comorbidity), dependent on the burden of OA as described by a set of variables $X$.

The objective of the methodology described in this appendix is to break down the observed combined utility $U_{inc}$ into the separate OA-related utility $U_{\text{OA}}\left( X \right)$ and the utility $U_{ex}$ without OA.

1. The multiplicative model to combine utilities

There are different ‘theories’ or approaches in the literature regarding how two utility values for separate conditions are combined in a person who experiences both conditions [1,2]. The recommended approach is to assume that utilities of separate conditions combine multiplicatively:

$$U_{inc}=U_{ex}\times U_{\text{OA}}\left( X \right)$$

For example, if OA-related utility is $U_{\text{OA}}\left( X \right)\text{ = 0.8}$ and utility without OA is $U_{ex}\text{=0.5}$, then the combined utility is $U_{inc}\text{=0.8×0.5=0.4}$. Here we consider the reverse problem: if the combined utility $U_{inc}$ is known and the OA-related utility $U_{\text{OA}}(X)$ can be estimated, then what would be the utility $U_{ex}$ without OA? This can be addressed in two parts: first, inverting the above multiplicative formula to solve for $U_{ex}$ and, second, obtaining a satisfactory estimate of $U_{\text{OA}}\left( X \right)$.

1. Inverting the multiplicative model

The defining formula above for the multiplicative model can be inverted as:

$$U_{ex}=\frac{U_{inc}}{U_{\text{OA}}\left( X \right)}$$

For example, if OA-related utility is $U_{\text{OA}}\left( X \right)\text{ = 0.7}$ and the combined utility is $U_{inc}\text{=0.6}$ then the utility without OA must be equal to $U_{ex}={U_{inc}}/{U_{\text{OA}}\left( X \right)}\text{= }\text{0.6/0.7 = 0.86}$. In practice, there are some issues with this formula:

- Utility $U_{ex}$ is undefined when $U_{\text{OA}}\left( X \right)$ is zero, because then the combined utility $U_{inc}$ is always equal to zero regardless the value of $U_{ex}$. The formula cannot be evaluated due to division by zero.
- Negative utility scores reflect health states worse than dead. For negative utilities, the multiplicative approach can give counterintuitive results. For example, if $U_{inc}$=‑0.3 and $U_{\text{OA}}\left( X \right)$=0.5, then $U_{ex}\text{=}\text{0.3/0.5=}\text{0.6}$. Here, the combined utility is better than separately for the non-OA conditions (i.e. $U_{inc}>U_{ex}$), as if the presence of the OA condition alleviates those non-OA conditions.
- The OA-related utility $U_{\text{OA}}\left( X \right)$ is estimated in a separate procedure and may turn out lower than the combined utility $U_{inc}$. For example, if $U_{inc}$=0.6 and $U_{\text{OA}}(X)$=0.4, then $U_{ex}\text{=0.6/0.4=1.5}$. This utility without OA is larger than 1, indicating health that is better than perfect. This is theoretically not possible.

We resolved the latter issue by simply truncating to at most 1. The first two issues with zero and negative values we resolved by rescaling utilities linearly to the positive range and back:

$$U^{*}=1-\left( 1-U \right)/\left( 1-M \right)$$

$$U=1-\left( 1-U^{*} \right)\times\left( 1-M \right)$$

Here M is a negative value slightly below the smallest possible utility value (e.g. $M\text{=-0.33<-0.329}$ for the three-level Dutch EQ-5D). If combined utility is multiplicative in the rescaled utility then, together with the truncation to 1, the inversion formula is:

$$U_{ex}=\text{min}\left\{ 1;M+\left( 1-M \right)\frac{U_{inc}-M}{U_{\text{OA}}\left( X \right)-M} \right\}$$

1. OA-related utility

Our multiplicative approach includes a value for the OA-related utility $U_{\text{OA}}(X)$, which requires to distinguish the OA and non-OA impact on utility. Consider a set of explanatory variables:
 $X$ = variables as is, including the impact of OA
 $X^{*}$ = same variables as they would have been without the impact of OA
For $X^{*}$ to be well defined, the set of variables $X$ should include only variables that are either not influenced by OA or entirely influenced by OA. For example, the demographic variables in $X$ and $X^{*}$ are identical, as they are not influenced by OA. On the other hand, for variables indicating the severity of OA, that severity would reduce to nil in $X^{*}$. This includes most of the questionnaire items of the Hip/Knee Disability and Osteoarthritis Outcome Score (HOOS-PS/KOOS-PS) and the Oxford Hip/Knee Score (OHS/OKS), for THAs/TKAs respectively. To avoid overestimating the burden of OA, we excluded items that did not specifically attribute symptoms to OA (e.g. “Could you do the household shopping on your own?”). As a result, non-specific OA burden is only included to the extent that it correlates with the specific variables.

To model the relationship between EQ-5D utility and the set of variables $X$ we used response mapping [3]. Respondents in our data set reported their EQ-5D domain levels $D$, from which we calculated the overall utility score using the Dutch tariff for the EQ‑5D:

$$U_{inc}=U_{\text{EQ-5D}}\left( D \right)$$

In response mapping, not these utilities but the probabilities of the domain levels are modeled:

$$p_{d}\left( x \right)=\text{Prob}\left\{ D=d | X=x \right\}$$

Using ordered probit models, we estimated this distribution over the possible domain levels, from which we then estimated the expected utility with and without OA:

$$V\left( X \right)=\sum_{d} p_{d}\left( X \right)\times U_{\text{EQ-5D}}\left( d \right)$$

$$V\left( X^{*} \right)=\sum_{d} p_{d}\left( X_{i}^{*} \right)\times U_{\text{EQ-5D}}\left( d \right)$$

By evaluating at the variables $X^{*}$ without the impact of OA, this $V\left( X^{*} \right)$ is an estimate for the expected utility without OA, but it lacks the variation in comorbidity. For the secondary analyses using the EQ‑VAS as the measure of health status, similar estimates for the expected utility with and without OA are obtained using a linear regression model on $\ln\left( {1-\text{EQ}\text{VAS}}/{100} \right)$ instead of the mapping.

The OA-related utility is finally found by again assuming a multiplicative utility model for utility with and without OA:

$$U_{\text{OA}}\left( X \right)=\frac{V\left( X \right)}{V\left( X^{*} \right)}$$

For someone without OA burden this OA-related utility equals one, since $U_{\text{OA}}\left( X^{*} \right)={V\left( X^{*} \right)}/{V\left( X^{*} \right)}=1$.

References

[1] Ara R, Brazier J. Estimating Health State Utility Values for Comorbidities. Pharmacoeconomics 2017;35:89–94. https://doi.org/10.1007/s40273-017-0551-z.

[2] Ara R, Wailoo AJ. Estimating health state utility values for joint health conditions: A conceptual review and critique of the current evidence. Medical Decision Making 2013;33:139–53. https://doi.org/10.1177/0272989X12455461.

[3] Hernández Alava M, Wailoo A, Wolfe F, Michaud K. A Comparison of direct and indirect methods for the estimation of health utilities from clinical outcomes. Medical Decision Making 2014;34:919–30. https://doi.org/10.1177/0272989X13500720.

# Table 1. Percentages of missing/imputed data for PROMS

|  | THA | | TKA | |
| --- | --- | --- | --- | --- |
| Variable | Complete cases | Percent imputed | Complete cases | Percent imputed |
| SES | 161,076 | 28% | 141,403 | 29% |
| EQ-5D (t0) | 72,611 | 68% | 52,775 | 73% |
| EQ-5D (t12) | 45,436 | 80% | 33,901 | 83% |
| EQ-VAS (t0) | 74,411 | 67% | 53,907 | 73% |
| EQ-VAS (t12) | 51,527 | 77% | 38,164 | 81% |
| OHS/OKS (t0) | 66,930 | 70% | 47,000 | 76% |
| OHS/OKS (t12) | 46,483 | 79% | 33,948 | 83% |
| HOOS/KOOS (t0) | 70,274 | 69% | 53,040 | 73% |
| HOOS/KOOS (t12) | 47,627 | 79% | 36,943 | 81% |
| SES: socioeconomic status, EQ-5D: EuroQol 5 Dimensions, EQ-VAS: EuroQol Visual Analogue Scale, OHS/OKS: Oxford hip/knee score, HOOS/KOOS: hip/knee disability and osteoarthritis outcome score, t0: baseline, t12: at 12 months post-procedure | | | | |

# Table 2. Five and eleven-year cumulative relative survival after THA and TKA, by sex and age group

|  |  |  | 5 year results | | | | 11 year results | | | |
| --- | --- | --- | --- | --- | --- | --- | --- | --- | --- | --- |
|  | Age group | N | Observed survival | Expected survival | Relative survival ratio (95% CI) | P-value | Observed survival | Expected survival | Relative survival ratio (95% CI) | P-value |
| **THAs** |  | 224777 | 0.919 | 0.870 | 1.057 (1.055-1.058) | <0.001 | 0.734 | 0.676 | 1.087 (1.080-1.094) | <0.001 |
| Males | Overall | 77273 | 0.906 | 0.859 | 1.054 (1.051-1.057) | <0.001 | 0.709 | 0.662 | 1.071 (1.057-1.084) | <0.001 |
|  | 18-50 | 4033 | 0.987 | 0.990 | 0.997 (0.992-1.001) | 0.188 | 0.957 | 0.969 | 0.987 (0.965-1.002) | 0.174 |
|  | 51-65 | 24821 | 0.970 | 0.957 | 1.014 (1.012-1.017) | <0.001 | 0.890 | 0.877 | 1.015 (1.002-1.027) | 0.018 |
|  | 66-80 | 40997 | 0.896 | 0.846 | 1.059 (1.055-1.063) | <0.001 | 0.652 | 0.595 | 1.097 (1.074-1.120) | <0.001 |
|  | 81+ | 7422 | 0.699 | 0.537 | 1.302 (1.278-1.325) | <0.001 | 0.273 | 0.147 | 1.860 (1.659-2.065) | <0.001 |
| Females | Overall | 147504 | 0.926 | 0.875 | 1.058 (1.056-1.060) | <0.001 | 0.747 | 0.683 | 1.094 (1.085-1.102) | <0.001 |
|  | 18-50 | 4450 | 0.990 | 0.992 | 0.998 (0.994-1.001) | 0.274 | 0.958 | 0.976 | 0.982 (0.956-0.998) | 0.097 |
|  | 51-65 | 37509 | 0.975 | 0.968 | 1.008 (1.006-1.009) | <0.001 | 0.916 | 0.909 | 1.009 (1.000-1.016) | 0.027 |
|  | 66-80 | 84791 | 0.934 | 0.890 | 1.050 (1.048-1.052) | <0.001 | 0.748 | 0.682 | 1.097 (1.085-1.108) | <0.001 |
|  | 81+ | 20754 | 0.792 | 0.623 | 1.270 (1.260-1.281) | <0.001 | 0.398 | 0.215 | 1.846 (1.761-1.932) | <0.001 |
| **TKAs** |  | 198689 | 0.933 | 0.885 | 1.054 (1.053-1.056) | <0.001 | 0.762 | 0.705 | 1.081 (1.074-1.088) | <0.001 |
| Males | Overall | 71768 | 0.920 | 0.874 | 1.052 (1.049-1.055) | <0.001 | 0.746 | 0.687 | 1.085 (1.072-1.098) | <0.001 |
|  | 18-50 | 2182 | 0.990 | 0.988 | 1.001 (0.995-1.005) | 0.595 | 0.968 | 0.965 | 1.004 (0.989-1.014) | 0.542 |
|  | 51-65 | 26984 | 0.970 | 0.956 | 1.014 (1.011-1.016) | <0.001 | 0.898 | 0.875 | 1.026 (1.015-1.035) | <0.001 |
|  | 66-80 | 37755 | 0.904 | 0.851 | 1.062 (1.058-1.066) | <0.001 | 0.675 | 0.605 | 1.116 (1.092-1.139) | <0.001 |
|  | 81+ | 4847 | 0.722 | 0.549 | 1.317 (1.289-1.344) | <0.001 | 0.286 | 0.155 | 1.850 (1.618-2.088) | <0.001 |
| Females | Overall | 126921 | 0.940 | 0.891 | 1.055 (1.053-1.057) | <0.001 | 0.770 | 0.715 | 1.078 (1.069-1.087) | <0.001 |
|  | 18-50 | 3260 | 0.987 | 0.991 | 0.996 (0.991-0.999) | 0.059 | 0.950 | 0.973 | 0.975 (0.946-0.994) | 0.045 |
|  | 51-65 | 39195 | 0.980 | 0.968 | 1.012 (1.011-1.014) | <0.001 | 0.924 | 0.910 | 1.015 (1.007-1.023) | <0.001 |
|  | 66-80 | 70465 | 0.942 | 0.893 | 1.055 (1.052-1.057) | <0.001 | 0.754 | 0.690 | 1.093 (1.079-1.106) | <0.001 |
|  | 81+ | 14001 | 0.809 | 0.640 | 1.265 (1.253-1.277) | <0.001 | 0.409 | 0.229 | 1.787 (1.694-1.880) | <0.001 |

# Table 3. Socioeconomic status scores by sex and age, compared to Dutch general population mean

|  | **THA cohort** | | **TKA cohort** | |
| --- | --- | --- | --- | --- |
|  | Mean (SE) | Difference with general population (95% CI) | Mean (SE) | Difference with general population (95%CI) |
| Overall | -0.064 (0.002) | 0.074 (0.034 to 0.114) | -0.164 (0.002) | -0.026 (-0.066 to 0.014) |
| Males |  |  |  |  |
| 18-50 years | -0.026 (0.019) | 0.112 (0.057 to 0.166) | -0.218 (0.028) | -0.081 (-0.150 to -0.012) |
| 51-65 years | -0.009 (0.007) | 0.128 (0.086 to 0.170) | -0.146 (0.007) | -0.009 (-0.051 to 0.034) |
| 66-80 years | -0.032 (0.005) | 0.105 (0.064 to 0.147) | -0.119 (0.006) | 0.019 (-0.023 to 0.060) |
| 81-100 years | -0.097 (0.014) | 0.040 (-0.008 to 0.089) | -0.143 (0.018) | -0.005 (-0.059 to 0.048) |
| Overall males | -0.031 (0.004) | 0.107 (0.066 to 0.147) | -0.133 (0.004) | 0.004 (-0.037 to 0.045) |
| Females |  |  |  |  |
| 18-50 years | -0.031 (0.018) | 0.107 (0.053 to 0.161) | -0.218 (0.023) | -0.081 (-0.142 to -0.020) |
| 51-65 years | -0.047 (0.006) | 0.091 (0.049 to 0.132) | -0.194 (0.006) | -0.057 (-0.098 to -0.015) |
| 66-80 years | -0.082 (0.004) | 0.055 (0.015 to 0.096) | -0.172 (0.004) | -0.035 (-0.075 to 0.006) |
| 81-100 years | -0.148 (0.009) | -0.010 (-0.054 to 0.033) | -0.178 (0.011) | -0.040 (-0.086 to 0.005) |
| Overall females | -0.081 (0.003) | 0.057 (0.016 to 0.097) | -0.181 (0.003) | -0.043 (-0.084 to -0.003) |

# Table 4. Health status of patients compared to the general population in secondary analysis using patients’ 12-month EQ‑VAS to estimate their generic health, mean (SE)

|  | **THA cohort** | | **TKA cohort** | |
| --- | --- | --- | --- | --- |
|  | Generic health status | Difference compared to the general population | Generic health status | Difference compared to the general population |
| Overall | 81.13 (0.07) | 4.38** | 74.4 (0.10) | 4.42** |
| Males |  |  |  |  |
| 18-24 years | 79.31 (4.67) | -10.09* | 76.32 (13.6) | -6.63 |
| 25-34 years | 82.07 (1.85) | -3.13 | 80.13 (6.60) | 0.38 |
| 35-44 years | 83.99 (0.74) | -0.91 | 77.13 (1.60) | -1.38 |
| 45-54 years | 83.08 (0.31) | 0.78 | 76.58 (0.44) | 0.83 |
| 55-64 years | 82.51 (0.18) | 2.21 | 76.11 (0.23) | 2.52 |
| 65-74 years | 82.16 (0.15) | 5.46* | 75.96 (0.18) | 5.95* |
| 75+ years | 80.98 (0.18) | 2.38 | 74.43 (0.22) | 2.87 |
| Overall males | 81.96 (0.11) | 3.38** | 75.64 (0.15) | 3.9** |
| Females |  |  |  |  |
| 18-24 years | 79.37 (4.56) | -2.93 | 74.56 (9.95) | -1.53 |
| 25-34 years | 82.24 (1.79) | -1.76 | 77.12 (4.42) | -0.69 |
| 35-44 years | 82.19 (0.74) | -0.41 | 75.65 (1.28) | -0.34 |
| 45-54 years | 81.93 (0.29) | 2.33 | 75.03 (0.36) | 2.35 |
| 55-64 years | 81.56 (0.15) | 0.46 | 74.68 (0.21) | 0.69 |
| 65-74 years | 81.01 (0.12) | 1.91 | 74.06 (0.16) | 2.22 |
| 75+ years | 79.85 (0.12) | 10.05** | 72.48 (0.18) | 10.41** |
| Overall females | 80.69 (0.09) | 4.89** | 73.7 (0.13) | 4.71** |
| *p-value < 0.05, **p-value < 0.001 | | | | |

# Table 5. Health status of patients compared to the general population in secondary analysis using patients’ baseline EQ‑VAS to estimate their generic health, mean (SE)

|  | **THA cohort** | | **TKA cohort** | |  |
| --- | --- | --- | --- | --- | --- |
|  | Generic health status | Difference compared to the general population | Generic health status | Difference compared to the general population |  |
| Overall | 78.10 (0.09) | 1.04 | 79.76 (0.09) | 2.32** |  |
| Males |  |  |  |  |  |
| 18-24 years | 76.10 (4.53) | -13.30** | 78.86 (14.91) | -10.54 |  |
| 25-34 years | 77.62 (2.01) | -7.58** | 79.12 (6.05) | -6.08 |  |
| 35-44 years | 77.71 (0.78) | -7.19** | 79.24 (1.30) | -5.66** |  |
| 45-54 years | 78.23 (0.30) | -4.07** | 79.56 (0.36) | -2.74* |  |
| 55-64 years | 78.55 (0.24) | -1.75 | 80.13 (0.19) | -0.17 |  |
| 65-74 years | 79.33 (0.16) | 2.63 | 80.84 (0.19) | 4.14 |  |
| 75+ years | 79.15 (0.19) | 0.55 | 80.75 (0.21) | 2.15 |  |
| Overall males | 78.98 (0.14) | 0.31 | 80.51 (0.14) | 1.90 |  |
| Females |  |  |  |  |  |
| 18-24 years | 76.40 (4.08) | -5.90 | 76.76 (9.58) | -5.54 |  |
| 25-34 years | 74.12 (2.22) | -9.88** | 77.48 (4.60) | -6.52 |  |
| 35-44 years | 75.63 (0.86) | -6.97** | 77.75 (1.07) | -4.85* |  |
| 45-54 years | 76.19 (0.30) | -3.41** | 78.26 (0.28) | -1.34 |  |
| 55-64 years | 77.35 (0.17) | -3.75* | 78.95 (0.16) | -2.15 |  |
| 65-74 years | 77.93 (0.12) | -1.17 | 79.51 (0.14) | 0.41 |  |
| 75+ years | 77.76 (0.15) | 7.96** | 79.65 (0.15) | 9.85** |  |
| Overall females | 77.65 (0.11) | 1.43 | 79.34 (0.11) | 2.56* |  |
| *p-value < 0.05, **p-value < 0.001 | | | | | |

# Table 6. Health status of patients compared to the general population in secondary analysis using patients’ baseline EQ‑5D to estimate their generic health, mean (SE)

|  | **THA cohort** | | **TKA cohort** | |  |
| --- | --- | --- | --- | --- | --- |
|  | Generic health status | Difference compared to the general population | Generic health status | Difference compared to the general population |  |
| Overall | 0.783 (0.001) | -0.083** | 0.794 (0.001) | -0.076** |  |
| Males |  |  |  |  |  |
| 18-24 years | 0.761 (0.063) | -0.215** | 0.756 (0.204) | -0.220 |  |
| 25-34 years | 0.742 (0.028) | -0.203** | 0.780 (0.088) | -0.165 |  |
| 35-44 years | 0.772 (0.010) | -0.181** | 0.782 (0.019) | -0.171** |  |
| 45-54 years | 0.789 (0.004) | -0.113** | 0.790 (0.005) | -0.112** |  |
| 55-64 years | 0.805 (0.002) | -0.108** | 0.807 (0.002) | -0.106** |  |
| 65-74 years | 0.808 (0.002) | -0.070** | 0.820 (0.002) | -0.058* |  |
| 75+ years | 0.795 (0.002) | -0.115** | 0.819 (0.003) | -0.091** |  |
| Overall males | 0.801 (0.001) | -0.097** | 0.814 (0.001) | -0.084** |  |
| Females |  |  |  |  |  |
| 18-24 years | 0.746 (0.052) | -0.179** | 0.700 (0.145) | -0.225 |  |
| 25-34 years | 0.685 (0.030) | -0.222** | 0.758 (0.055) | -0.149* |  |
| 35-44 years | 0.734 (0.010) | -0.183** | 0.746 (0.016) | -0.171** |  |
| 45-54 years | 0.755 (0.004) | -0.122** | 0.755 (0.004) | -0.122** |  |
| 55-64 years | 0.774 (0.002) | -0.092* | 0.773 (0.002) | -0.093* |  |
| 65-74 years | 0.782 (0.001) | -0.112** | 0.789 (0.002) | -0.105** |  |
| 75+ years | 0.768 (0.002) | -0.019 | 0.788 (0.002) | 0.001 |  |
| Overall females | 0.774 (0.001) | -0.076** | 0.783 (0.001) | -0.071** |  |
| *p-value < 0.05, **p-value < 0.001 | | | | | |

# Table 7. Proportions of survival difference explained by calibration of SES and generic health values to general population norms, in secondary analyses using patients 12-month EQ‑VAS to estimate generic health

|  | **THA cohort** | | | | **TKA cohort** | | | |  |
| --- | --- | --- | --- | --- | --- | --- | --- | --- | --- |
|  | Difference between ${LE}_{\text{LROI}}$ and ${LE}_{\text{NL}}$  (years) | EPD by SES | EPD by Health | EPD by SES & Health | Difference between ${LE}_{\text{LROI}}$ and ${LE}_{\text{NL}}$ (years) | EPD by SES | EPD by Health | EPD by SES & Health |  |
| Overall | 1.77 | 1.81% | 5.97% | 7.77% | 0.66 | 0.00% | 12.47% | 11.72% |  |
| Males |  |  |  |  |  |  |  |  |  |
| 18-50 years | -0.11 | 0.00% | 0.00% | 0.00% | -2.03 | 0.00% | 0.00% | 0.00% |  |
| 51-65 years | 1.08 | 5.40% | 9.35% | 14.73% | -0.1 | 0.00% | 0.00% | 0.00% |  |
| 66-80 years | 1.61 | 2.52% | 8.02% | 10.52% | 1.27 | 0.69% | 8.74% | 9.42% |  |
| 81-100 years | 1.93 | 0.70% | 2.08% | 2.78% | 2.17 | 0.13% | 1.98% | 2.09% |  |
| Overall males | 1.38 | 3.22% | 7.64% | 10.85% | 0.72 | 0.44% | 13.05% | 13.47% |  |
| Females |  |  |  |  |  |  |  |  |  |
| 18-50 years | 0.02 | 66.04% | 65.79% | 69.75% | -3.15 | 0.00% | 0.00% | 0.00% |  |
| 51-65 years | 1.34 | 3.03% | 2.38% | 5.40% | -0.69 | 0.00% | 0.00% | 0.00% |  |
| 66-80 years | 2.19 | 1.06% | 5.73% | 6.78% | 1.16 | 0.00% | 7.94% | 7.40% |  |
| 81-100 years | 2.67 | 0.06% | 6.44% | 6.50% | 2.47 | 0.00% | 5.49% | 5.26% |  |
| Overall females | 1.98 | 1.29% | 5.36% | 6.64% | 0.62 | 0.00% | 12.08% | 10.57% |  |
| EPD: Explained percentage of survival difference. SES: socioeconomic status. ${LE}_{\text{LROI}}$: life expectancy of patients without calibration for SES or health. ${LE}_{\text{NL}}$: life expectancy according to general population lifetables. | | | | | | | | | |

# Table 8. Proportions of survival difference explained by calibration of SES and generic health values to general population norms, in secondary analyses using patients baseline EQ‑5D to estimate generic health

|  | **THA cohort** | | | | **TKA cohort** | | | |  |
| --- | --- | --- | --- | --- | --- | --- | --- | --- | --- |
|  | Difference between ${LE}_{\text{LROI}}$ and ${LE}_{\text{NL}}$  (years) | EPD by SES | EPD by Health | EPD by SES & Health | Difference between ${LE}_{\text{LROI}}$ and ${LE}_{\text{NL}}$ (years) | EPD by SES | EPD by Health | EPD by SES & Health |  |
| Overall | 1.76 | 1.77% | 0.00% | 0.00% | 0.65 | 0.00% | 0.00% | 0.00% |  |
| Males |  |  |  |  |  |  |  |  |  |
| 18-50 years | -0.23 | 0.00% | 0.00% | 0.00% | -2.08 | 0.00% | 0.00% | 0.00% |  |
| 51-65 years | 1.06 | 5.33% | 0.00% | 0.00% | -0.12 | 0.00% | 0.00% | 0.00% |  |
| 66-80 years | 1.61 | 2.44% | 0.00% | 0.00% | 1.26 | 0.68% | 0.00% | 0.00% |  |
| 81-100 years | 1.92 | 0.69% | 0.00% | 0.00% | 2.16 | 0.13% | 0.00% | 0.00% |  |
| Overall males | 1.37 | 3.16% | 0.00% | 0.00% | 0.70 | 0.45% | 0.00% | 0.00% |  |
| Females |  |  |  |  |  |  |  |  |  |
| 18-50 years | -0.10 | 0.00% | 0.00% | 0.00% | -3.19 | 0.00% | 0.00% | 0.00% |  |
| 51-65 years | 1.31 | 3.01% | 0.00% | 0.00% | -0.71 | 0.00% | 0.00% | 0.00% |  |
| 66-80 years | 2.20 | 1.03% | 0.00% | 0.00% | 1.16 | 0.00% | 0.00% | 0.00% |  |
| 81-100 years | 2.66 | 0.06% | 0.00% | 0.00% | 2.47 | 0.00% | 0.06% | 0.00% |  |
| Overall females | 1.97 | 1.81% | 0.00% | 0.00% | 0.61 | 0.00% | 0.00% | 0.00% |  |
| EPD: Explained percentage of the survival difference. SES: socioeconomic status. ${LE}_{\text{LROI}}$: life expectancy of patients without calibration for SES or health. ${LE}_{\text{NL}}$: life expectancy according to general population lifetables. | | | | | | | | | |

# Table 9. Proportions of survival difference explained by calibration of SES and generic health values to general population norms, in secondary analyses using patients baseline EQ‑VAS to estimate generic health

|  | **THA cohort** | | | | **TKA cohort** | | | |  |
| --- | --- | --- | --- | --- | --- | --- | --- | --- | --- |
|  | Difference between ${LE}_{\text{LROI}}$ and ${LE}_{\text{NL}}$  (years) | EPD by SES | EPD by Health | EPD by SES & Health | Difference between ${LE}_{\text{LROI}}$ and ${LE}_{\text{NL}}$ (years) | EPD by SES | EPD by Health | EPD by SES & Health |  |
| Overall | 1.75 | 1.81% | 0.95% | 2.75% | 0.64 | 0.00% | 5.57% | 4.81% |  |
| Males |  |  |  |  |  |  |  |  |  |
| 18-50 years | -0.19 | 0.00% | 0.00% | 0.00% | -2.08 | 0.00% | 0.00% | 0.00% |  |
| 51-65 years | 1.04 | 5.55% | 0.00% | 0.53% | -0.12 | 0.00% | 0.00% | 0.00% |  |
| 66-80 years | 1.60 | 2.51% | 4.08% | 6.59% | 1.26 | 0.69% | 5.91% | 6.58% |  |
| 81-100 years | 1.93 | 0.70% | 0.95% | 1.65% | 2.17 | 0.13% | 1.77% | 1.88% |  |
| Overall males | 1.36 | 3.25% | 0.52% | 3.75% | 0.70 | 0.45% | 5.55% | 5.98% |  |
| Females |  |  |  |  |  |  |  |  |  |
| 18-50 years | -0.07 | 0.00% | 0.00% | 0.00% | -3.18 | 0.00% | 0.00% | 0.00% |  |
| 51-65 years | 1.30 | 3.10% | 0.00% | 0.00% | -0.71 | 0.00% | 0.00% | 0.00% |  |
| 66-80 years | 2.18 | 1.06% | 2.46% | 3.51% | 1.15 | 0.00% | 4.88% | 4.34% |  |
| 81-100 years | 2.66 | 0.06% | 5.68% | 5.73% | 2.47 | 0.00% | 5.13% | 4.91% |  |
| Overall females | 1.96 | 1.86% | 0.93% | 2.79% | 0.61 | 0.00% | 5.58% | 4.05% |  |
| EPD: Explained percentage of the survival difference. SES: socioeconomic status. ${LE}_{\text{LROI}}$: life expectancy of patients without calibration for SES or health. ${LE}_{\text{NL}}$: life expectancy according to general population lifetables. | | | | | | | | | |

# Figure 1A. Predicted and expected survival of female patients after THA procedure


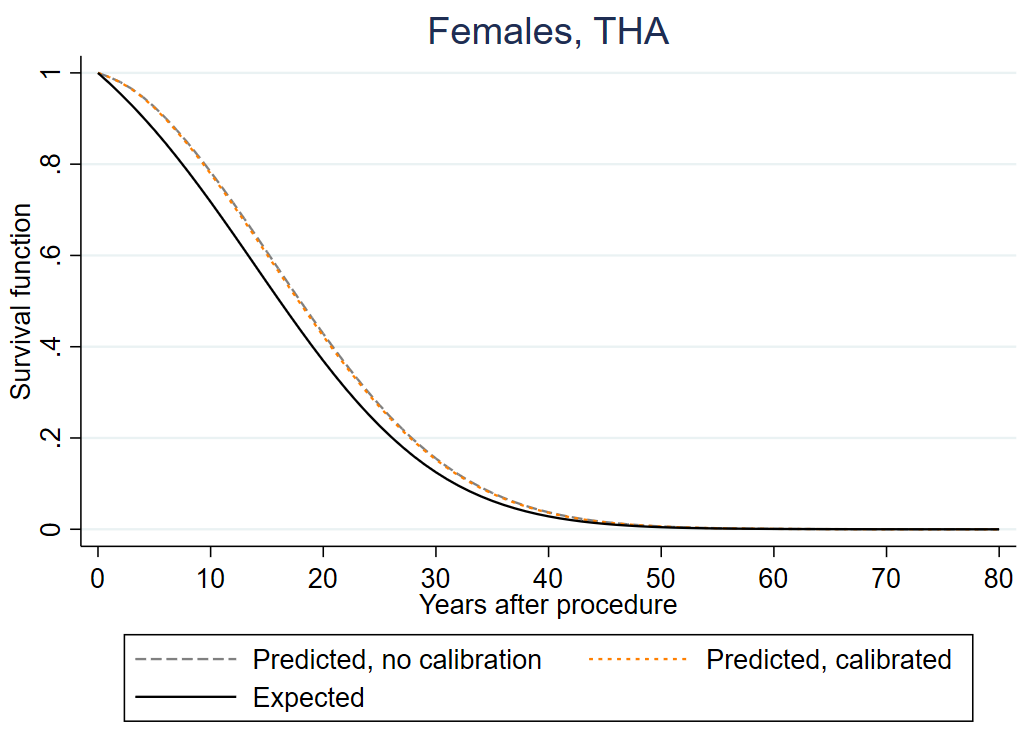


# Figure 1B. Predicted and expected survival of male patients after THA procedure


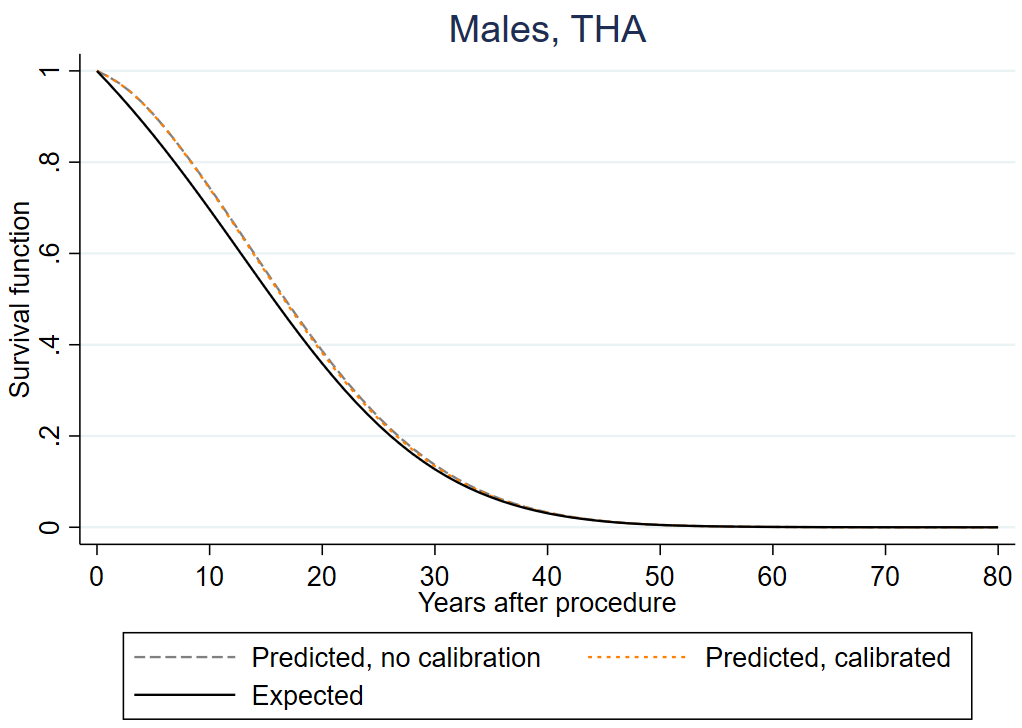


# Figure 1C. Predicted and expected survival of male patients after TKA procedure


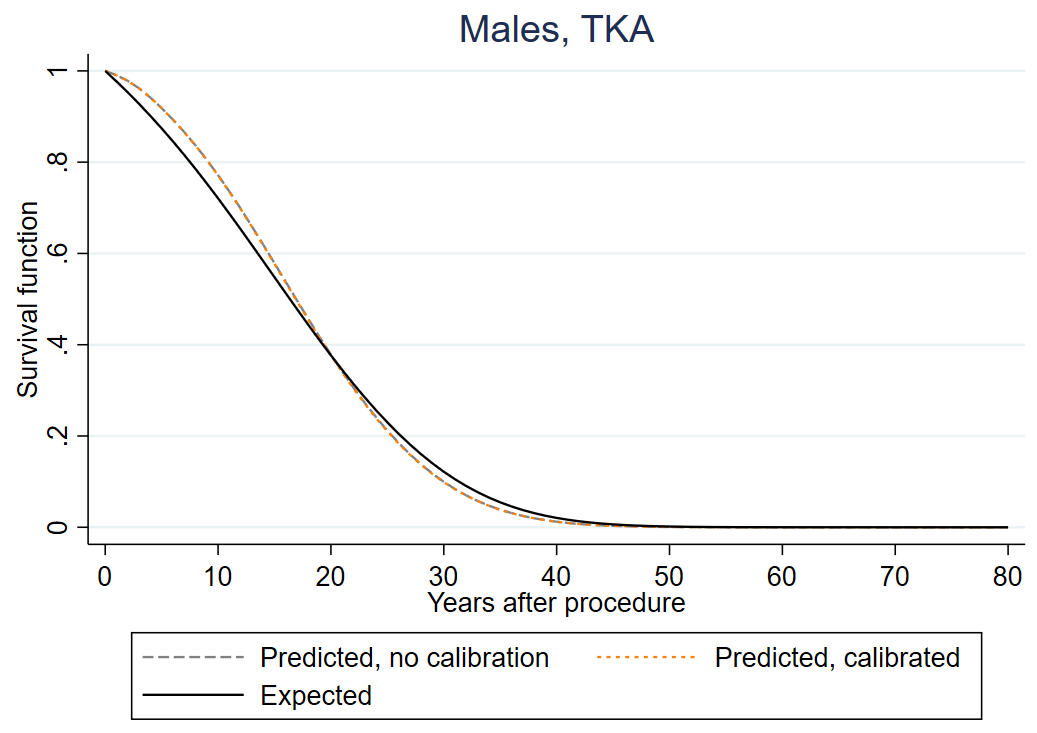


# Figure 1D. Predicted and expected survival of female patients after TKA procedure


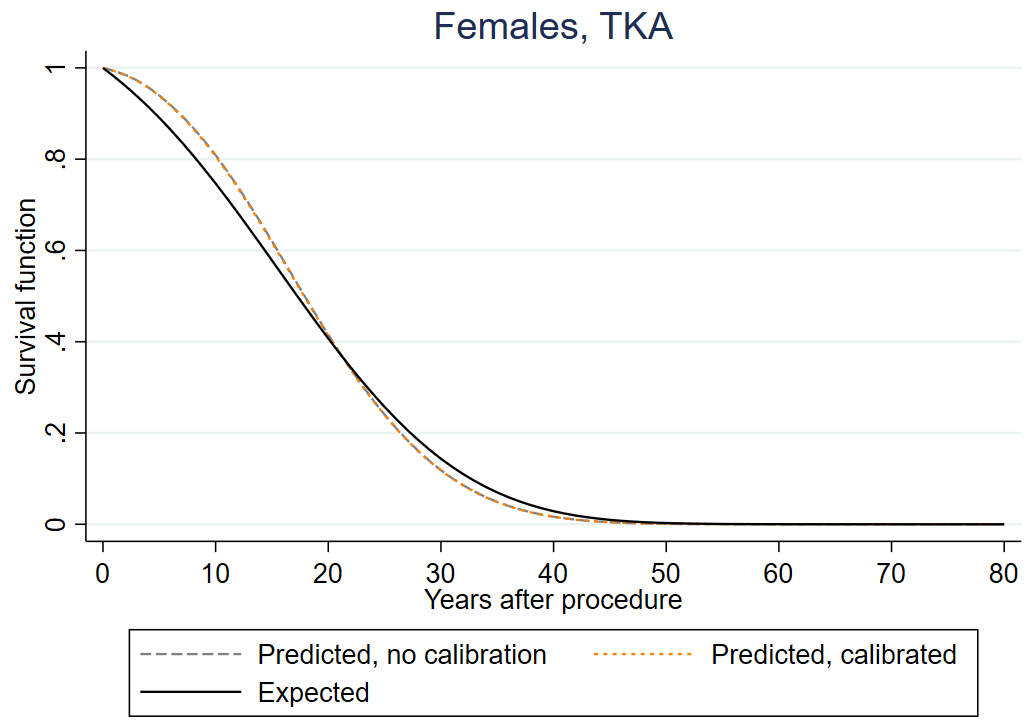


# Figure 2A. Standardized mortality ratio (SMR) over follow up (in years) among TKA patients


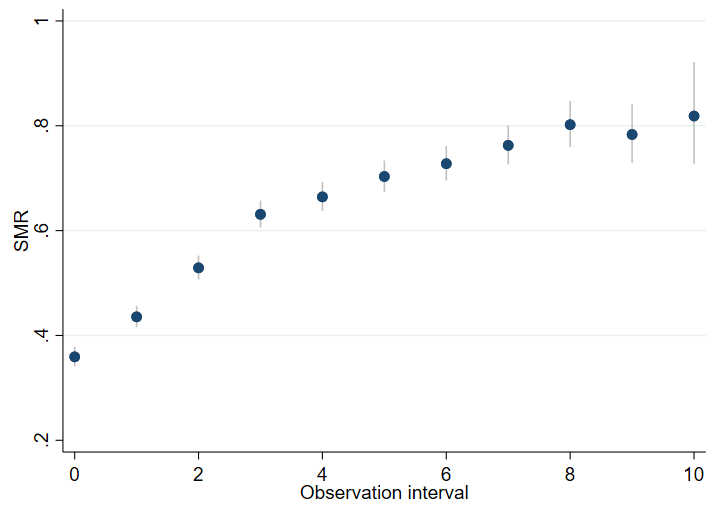


# Figure 2B. Standardized mortality ratio (SMR) over follow up (in years) among THA patients


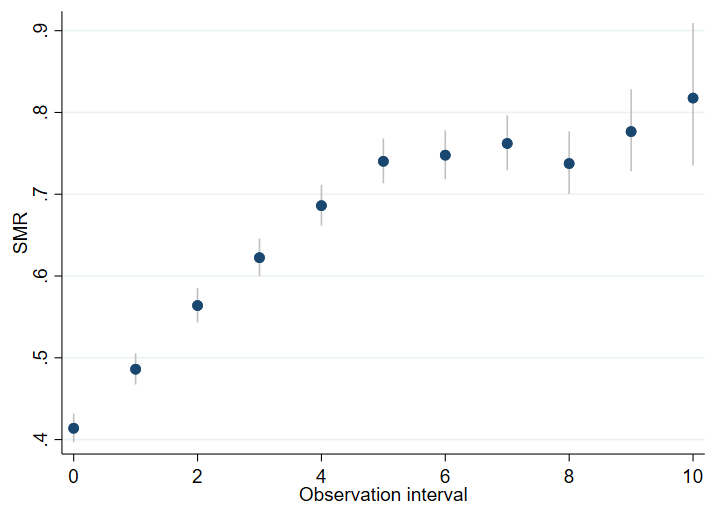

Supplement: Multimedia component 1 [file mmc1.docx]
